# Supplementary material for: Multiple-P450 Gene Co-Up-Regulation in the Development of Permethrin Resistance in the House Fly, Musca domestica
Source: Int J Mol Sci. 2023 Feb 6;24(4):3170. doi: 10.3390/ijms24043170 (PMC9959456; doi:10.3390/ijms24043170)
Supplement: Supplementary file 1 [file ijms-24-03170-s001.zip › ijms-2112179-SI.pdf]

Supplementary Materials

A.

CYP6A36

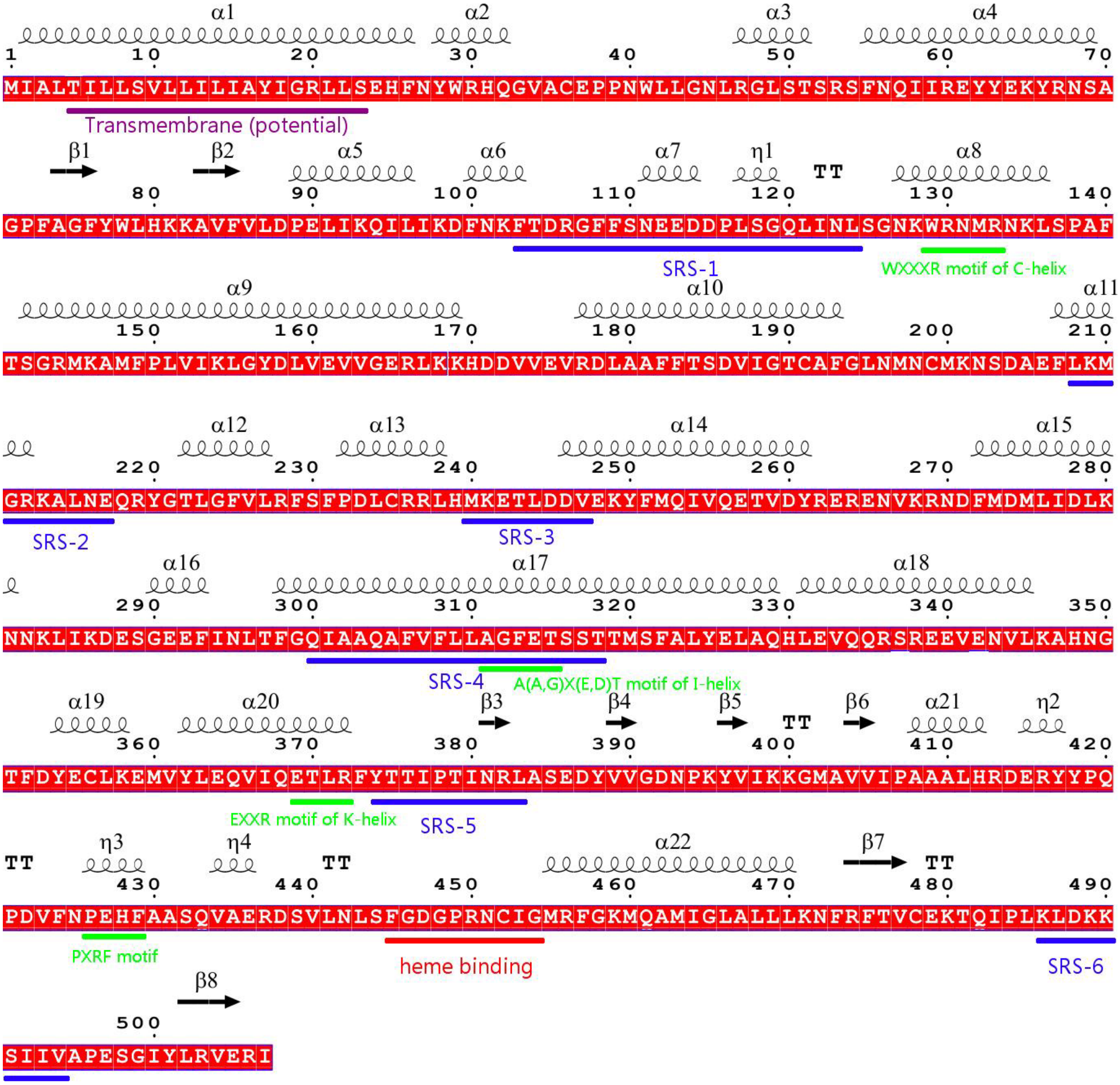

B.

CYP6D10

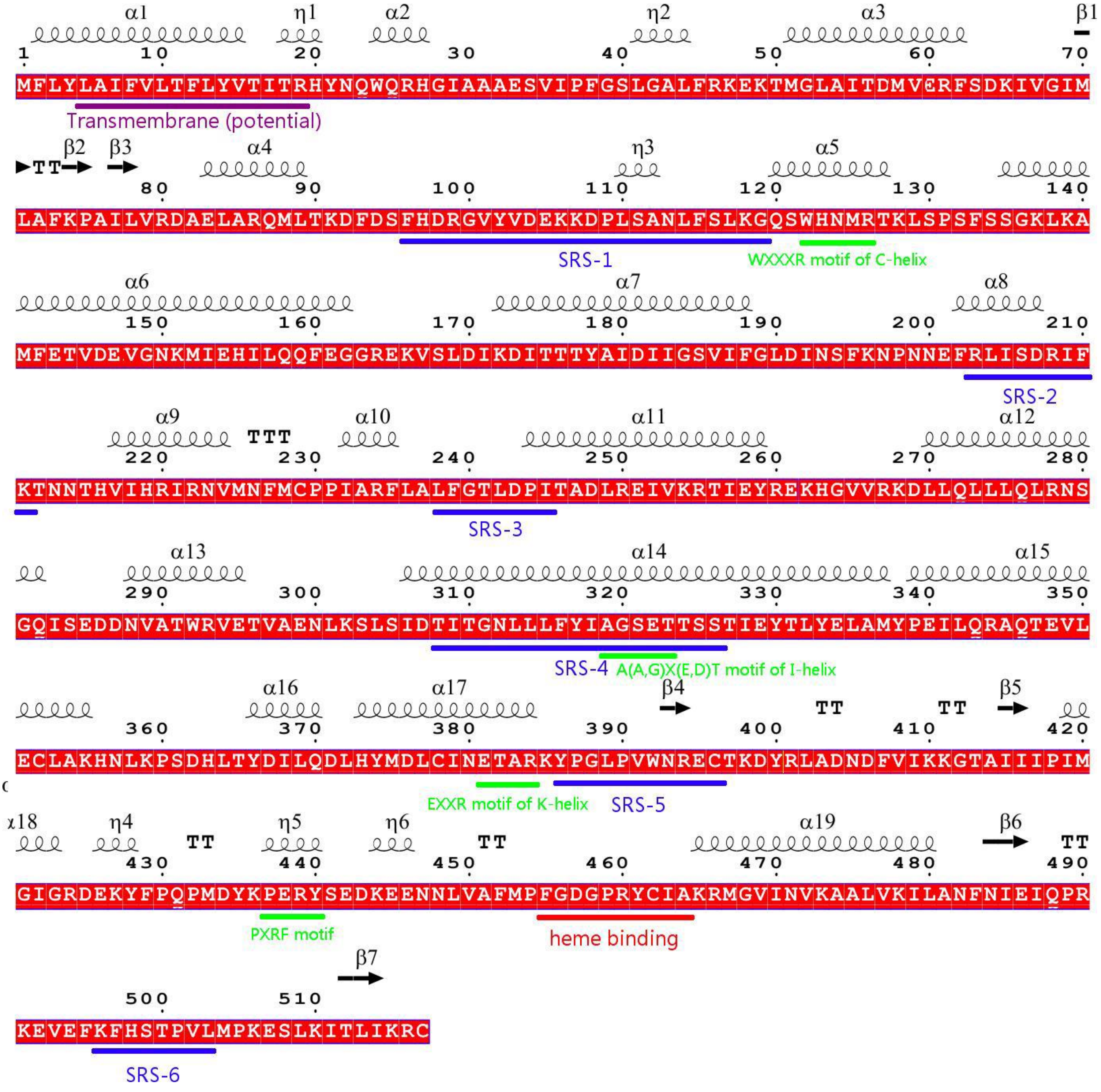

Transmembrane (potential)
SRS-1
WXXXR motif of C-helix
SRS-2
SRS-3
SRS-4
A(A,G)X(E,D)T motif of I-helix
EXXR motif of K-helix
SRS-5
PXRF motif
heme binding
SRS-6

C.

CYP4S24

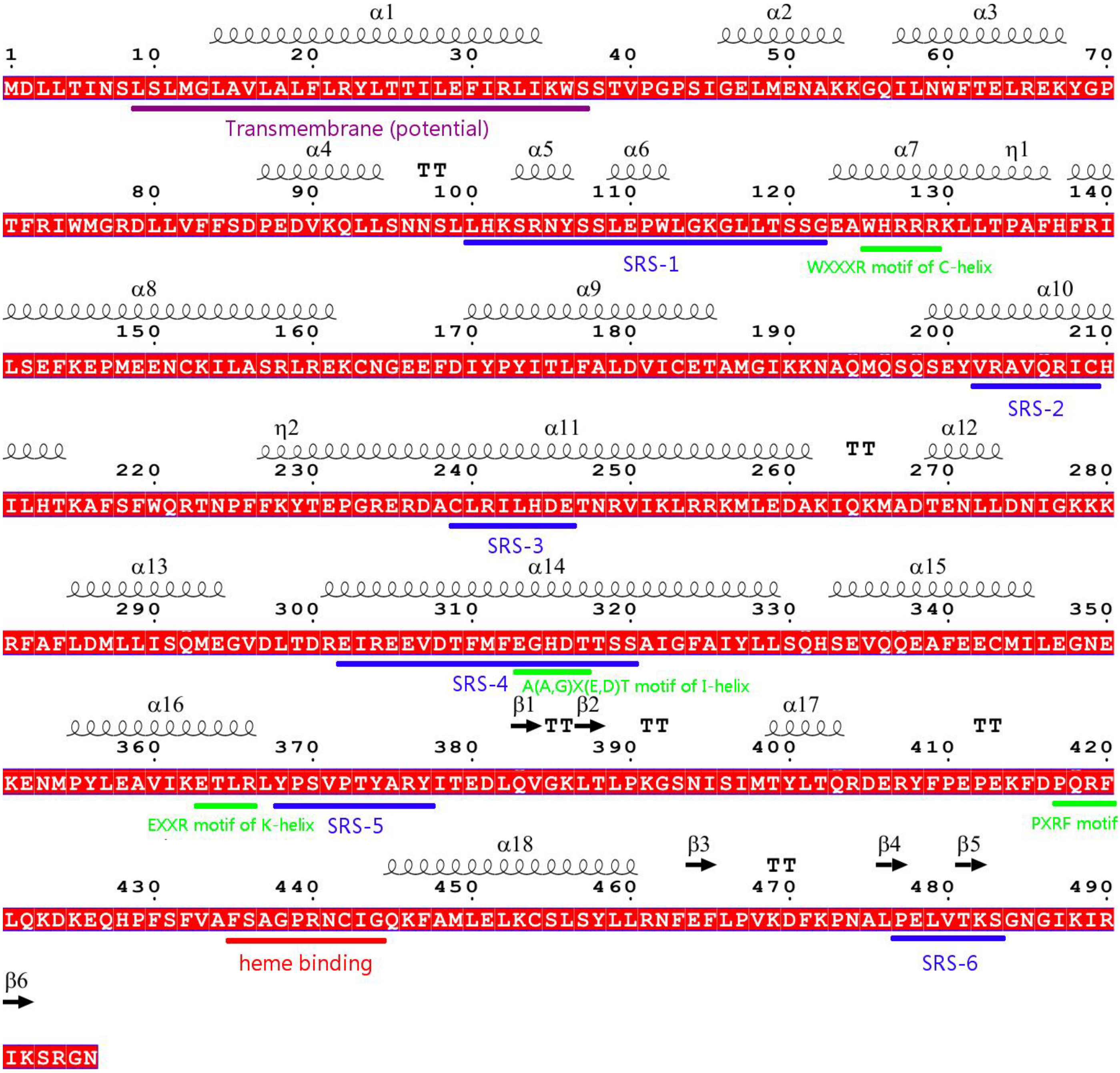

Figure S1. Deduced amino acid sequence of P450s. The sequence analysis was conducted by T-COFFEE (http://tcoffee.crg.cat/) and ESPript 3.0 (http://espript.ibcp.fr/ESPript/ESPript/) (Gouet et al. 2003). Alpha-helices, eta-helices, beta strands and strict beta turns are marked as α, η, β and TT, respectively. The predicted transmembrane region, conserved domains common to cytochrome P450s, and the proposed substrate recognition sites (SRS) are indicated. (A) CYP6A36. (B) CYP6D10. (C) CYP4S24.

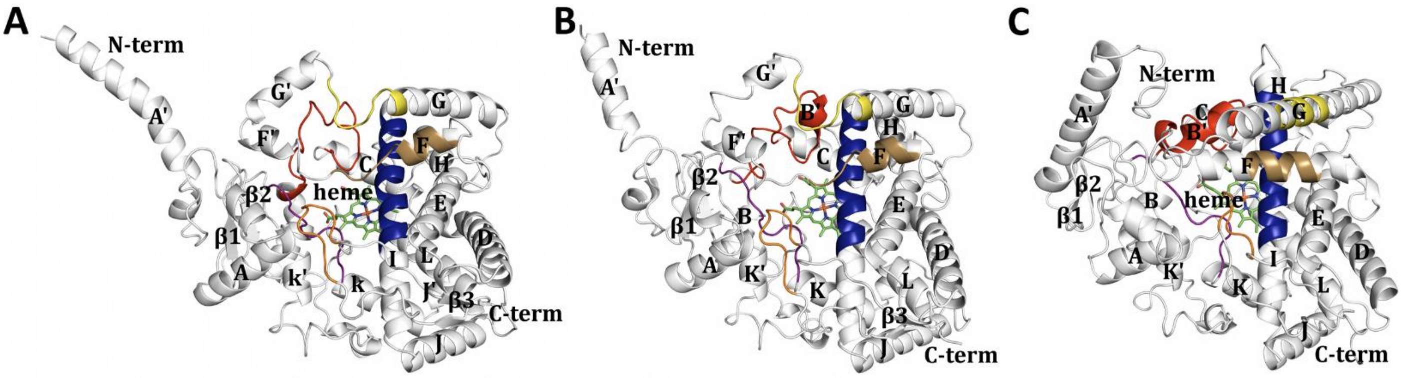

Figure S2. Topology of P450s. Secondary structure of helices and sheets are labeled. Six putative SRSs based on Gotoh's predicted models (Gotoh 1992) are shown. SRS 1 to 6 are shown in red, wheat, yellow, blue, purple and orange color, respectively. The heme group is represented by sticks. (A) CYP6A36. (B) CYP6D10. (C) CYP4S24.

Table S1. List and sequences of the primers used

Gene	Forward primer (5'-3')	Reverse primer (5'-3')
Primers used for quantitative real time PCR		
CYP18A1	TTAAGACCACCCTGCTGTGGATCA	GTGGCCAATGGTACAATGCTGGAA
CYP304A2	CAATGTTGTCGGCTCAGGTCGTTT	AAGGTACCAGCGTCTCAATTCGCA
CYP3073A1	AAACGACAGCGGAGGGTTTGAGAT	TAACCACACCATTGCTCAGTCGGA
CYP3073A2	GGTGCATGTATTTAGTGCGGCCTT	AGCTCGAACTCTCCGTCATCGTTT
CYP3073B1	ACAATGTTGGGTCACAATGCCAGG	TTCAATGGTCTCCCGACATTCCCT
CYP305A1	TCACAAACCACCAGCACCACAATG	TAGGCCTTGTTACCCAAACTGGGA
CYP306A1	TGCCATTCTATTCGCGATCGGTCA	GCACTGAAGGCCACAAAGCTGAAT
CYP28B1	TACACCACCGAAGTTGTCAGCGAT	TCATCACCTTCTTTAGGCTGGGCA
CYP28B2	GAAACCCGGAGACCCAAGAGAAAT	AGTTTAGAGTTCACAGCCACGGGT
CYP28G6	TACTGGGTCTTTCGGCTGATGCTT	AAACTCCTCCACGGGCTTTGGTAT
CYP310B2	GATAGCATCGACGGCAGTATTT	CTTTGTTTCACTGTTCGCCATTAT
CYP317A3	TATGCAGCAAAGGGTACGGGAAGA	AGAGTGCGACGCATTACATAGGGT
CYP6A1	GTCTACGCGAGGAGGTTAATG	GCGGAGTGTTTCATTCAATACC
CYP6A24	TTCGAATACCCAAGGGAACACCCA	AAATCGGGCGCCAATACAGTTTCG
CYP6A25	TTCCCGGTCATCCGAAATATG	CGTCGGGATTGGGATAATACTG
CYP6A36	TTTATCCTTTGGTGATGGTCCC	CGCCACAATGATGCTCTTCTTA
CYP6A37	ATGAGTTGGCCCAGAATCAGGAGA	ATTCAGAATGGGCAGGACGGTGTA
CYP6A4	AAGGGCATAGATTTGTCGCATGGC	CCTGGTGCCTAGCCAATTCATAGA
CYP6A40	TGAGGGAGAGCAAGCAAATC	TGTTGTGGAGGAGGTCTCATA
CYP6A52	ATGTGATTGGTCGCTGTGCCTTTG	TACTTCTCCACATCGGGCATGGTT
CYP6A7	GCATTGTCCGCGAAACTGTGGAAT	AACACAAAGGCCTGGCCAGTTATC
CYP6A54	GAAGGTATTCGGTTCGGAGAAA	TGGCTCCAAAGTAGCGTAAAT
CYP6A63P	AGGCCATGATGGAGATGGCCTATT	ATCCTCCATGCAGACACGCTGTAA
CYP6A57	GCTGCCCAGGTGTTTGTCTTCTTT	AAAGTCTCTTTGACCTCCTGGCGT
CYP6A58	AATCGCCAAGCTGTCGAAGACTAC	TCCGGATTGAAGACATTGGGTTGG
CYP6A59	CCTATGAGTCGCTGAAGGATATG	GGGACCTGATAGTCTTTGACAC
CYP6A5	CAAATGGCCGCTCAGACCTTTGTT	AGGCCCTCATAGGTTATTTCGCCA
CYP6C2	TCTGTGGCGTAAGGTGAGAACCAA	TGCCAATGACATCGGTGGTGAAAC
CYP6D10	ACTGCTCGCAAATATCCTGGCCTA	TCCGGCTTATAATCCATGGGCTGA
CYP6D11	CAGCGGCTACCATAGCATTTA	CCCTCAACTTATGTTCCGTCAA
CYP6D3	AACTGCCTCAAGTACCTCATTTA	TACGTCCATCCGGCTTTATTC
CYP6D8	TGGTCGGCGGTAAAGACAAAGGAT	CGGCATTGCGTGCCAATTCATAGA
CYP6EK2	CCTTTCCGAATTTGGCCCGGAAAT	GGCCGCTATTTGTTCCAACGTCAA
CYP6FS2	AACCTTTAGCAACTCTCTGCGGGA	ATTGTGTGTTGCGATAACGCACGG
CYP6FT3	GACGATGACAGGGATATGGATAAG	GGCCAATTCAAAGAGAGCATTT
CYP6G4	TGACTGCTGGCTTTGAGACATCCT	ATTTCTCGACGCAAACGTTCCTGC
CYP6GU1	TGCTCTCTATGAATTGGCCCGGAA	AGGACGGCATATTTGCGTAGGGTT
CYP6GW1	GTTCCAGTTGACCATGGATATGA	CTCATATAGGGCCTGACACAAAG
CYP6V3	CGCAAGTACCCGATTGTGCCATTT	AAAGAGGTCTGGTTCGGGCCAATA
CYP9F10	GTTCTACGCAAATGGCCGGTGAAT	AAACGTTCCGGATCAAAGGCACTG
CYP9F11	AAATGGTCGTGTCGGAGGTGTTGA	TTCCGGATCGAAGGCATCAGGATT
CYP9F12	CGATGAGGCCATGTCGTATT	CAAATGCCGTCGAAGCAATAA
CYP9F7	CGATCAACGACAACCAATGCTGCT	TGGCGACAAAGTGTTGCGCATATC

CYP9F8v1	ATGCGTTCCATGTTCCAGCTGATG	GGCCAAATGCAGTCGAGGCAATAA
CYP9F9	AAACAAGGCAAGGGAGAGGATGGA	GACTTGCAGACCAAATGCAGTGCT
CYP311A1	CCAATACCTCGGATCTCAACAA	AATGTTACGCCAGGTGGATAG
CYP313D1	GGCTTTCGATGTGACCTATGCCAA	TTGATGACACATTGCACACCAGCC
CYP313D2	TGCACAGACGCAAGGATATATGGG	TTAGCAACTTTGCCAAGGCCACCT
CYP318B1	TGCATCGTAGTGCAGATGTTTGGG	GCCAAGTACATGCTATAGCGGCTT
CYP438A4	CGGTCGATAAGGCAACGATAG	GGCATACTGATACAAAGCGAATG
CYP4AA1	AACACTGGGATAGAGTCGCAACGA	AGGCCATGATGGAGATGGCCTATT
CYP4AC6v1	GAGGAGTATCGCAAGAAGAAGG	CATCCAGCAGCGTATCCAA
CYP4AD1	GCCCTCTTCACCACAGATTTA	AATGCCACAGAGAAGTCGATAG
CYP4AE3	TTGCCTCTATGCACTATCGCGTCA	CCAACAGCCGGAATTGGTGGAAAT
CYP4C74	TGAGGAGGTTGACACGTTTATG	TCGACAACACGTTCCTGATATT
CYP4D3	TTCGGCCGTAACGTTCTGCTTCTA	GCAGCGTCTCCTTGATGCACAAAT
CYP4D36	GTCGAACCATTTACGCTGGCCAAA	ATTGTGTAGACGCCGGAACAATGC
CYP4D4	GCGAATGGCTTTCCTTGATGTGCT	TCGTGGTGTCATGTCCTTCGAACA
CYP4D54	CAGTTGCCGGGAATAACAACACCA	AGATGCGAACACTGCTCCCACATA
CYP4D55	GCCTTGTTGGATGTGCTATTG	TAAGGGCCTGCATCAAAGAG
CYP4D56	TGTTTCCAGTTGTGTGTCGCATGG	TTGGCGAGGTGAGTTTGAAGAGGA
CYP4D58v1	ATTTGCCACCGCCGAAATGAAGAG	ACCCAATTGGACATCAGTCTCGGA
CYP4D9	CAAGCGTTGCCGACATAATAG	TCCCTCATCATCTTGCGATAC
CYP4E10	GCACCACCCTGCTACTATTT	CTCGAAGGGATTGTAGGTCATT
CYP4E11	CAAGATCATAACACCGGCATTTC	GCCTCCCTCAATTTGTCCATA
CYP4E7	ATCAACATGCGTGCCTTCAATCCC	ATTCTTTGCCTCCTGCTTGGCTTC
CYP4G13	ATTGCCCGTAAAGCGGAAGAGGAT	TAGATGTCGGCACGACGATGAACA
CYP4G2	ATGACCCGTAAAGTGGTGACGGAA	TCATCCAAATCATCGCGCAAACCC
CYP4G99	TGAGACATCGTAGCCTCTTTCTG	CGTTGCCATTGGAGTTTAGAC
CYP4P8	AATGCGCGAAGCAGTCGAAGAAAC	AAAGCATACGGATGACGTCCCACA
CYP4P10	ACGACATACATCGTAATCCCAGGC	AAATGCAAACGGGTGCCTCTGTTC
CYP4S23	TGCCATTCTATTCGCGATCGGTCA	GCACTGAAGGCCACAAAGCTGAAT
CYP4S24	GGTCCAACATTTCGCATTTG	GCTTCACCACTACTCGTCAGCA
CYP12A1	AAGTGGAGTGCCAGAGAAAC	GTATCCACACCAGCCAATATCA
CYP12A3	CCCACAGCAAAGCGTATGTTCCAA	GCCGAACAATGGCCTCTTCAACAA
CYP12A14	AGGGCCTGCATTAAGGAATCGCTA	AGCTCTCAACCATCGTTCGGGTAA
CYP12A16	GCCAATGGCAACATCGAACCAAGT	AAGAGCAAGCCGGTAAAGGCAGAT
CYP12A17	TAAGGGCCTGCATCAAAGAG	GATAACCGCTGAGGACAACAT
CYP12A13	CCCTGCGCATGTATCCATTGACAT	ACTTCCTGATTCGGTTGGCCTCAA
CYP12A2	AGTGGCTATCGTGTTCCCAAAGGT	GGCATGTGGACATTCAGCGGATTT
CYP12G2	ATGGGCGGTGTAGAAATCACCTCA	ATGGAGAAATCTTCGTGCCGTCCA
CYP12G4	TGTTTGGCAATATGCGAGCCTTGG	TTTCGGGCTGTGATATCTTCGGCA
CYP301A1	ACGGATCGCCTAAAGGTTCAAGGA	CAAACCGCCATGGAAATGGTGTCA
CYP302A1	TGCTTGTCTGAAGGAGGTATTC	TCCTTCGGCACCAAATATCC
CYP314A1	ACCGAACAGCCGGAGAAGATCAAA	TTTCCTGTAGGTCTGCGTGGGAAA
CYP315A1	TTTATACTGCCGGTCGTGATCCCA	ATAGGGCCAATTTGCGGCCAATAC
Actin	ATGAGGCTCAGAGCAAACGTGGTA	AGTCATCTTCTCGCGATTGGCCTT
Dm RPL11	CGATCCCTCCATCGGTATCT	AACCACTTCATGGCATCCTC
Primers used for autosome mapping		
CYP4E10	F1: CGATTTGAAGAACCAGAAGC	R: ACTCATTGTTGTGGCTCTCA

	F2: AAGCTGATTAAGGCGGAAC	
CYP4E11	F1: CCAGGCCTGTAACGGCAATCC	R: ACTCATTGTTGTGGCTCTCA
	F2: GGCCGATAACTATGCCACCGTT	
CYP4G13	F1: GTCTTCAAGGATTGTGGTGAAAC	R: TATTGTCACGCTCACGTAGCTTG
	F2: TTTGTGCCCACATTTGTCAAG	
CYP4G99	F1: TTGGACTTGCTCTTGGAGA	R: TGGTAGCCACTGTGATGGT
	F2: TGGTGCCACCATTACGGACACT	
CYP4S24	F1: AGAGAATCTGCCACATCTTGC	R: TGCCGAACTTGTCGTATCG
	F2: CGAAGATGCCAAGATACAAAAT	
CYP6A36	F1:TTTAAGGGGTCTCTCGACGAGT	R: AATGGAAACATGGCCTTCATA
	F2:TTATGAGAAATATAGAAATTCCGCC	
CYP6A52	F1: ATGTGATTGGTCGCTGTGCCTTTG	R: CATACCCTTGCGTATGACATAG
	F2: CCCAAATCGGAATTCCGC	
CYP6A40	F1: CATTCTCACCAGGGATTTCA	R: AAAGGCACAGCAGCCAAT
	F2: GCTGACACCCACCTTCAGTTCA	
CYP6A58	F1: CACCTTTACCTCGGGCAAA	R: CGCCATCCTCACTCTTCATC
	F2: AACCGAAACCATGCCCGATGT	
CYP6D3	F1: GACGGCTACAAATCTCAAGG	R: GTTTCATCGGCAAATCGTTC
	F2: TGAATTGGCCATGAATCCAGAT	
CYP6D10	F1: CTACCCGTTTGGAATCGTG	R: TCCTTGCGTGGCTGTATT
	F2: AAAGCTGCCCTGGTAAAAATG	
CYP9F10	F1: GCTTTTCTTATTGCCGGCATAG	R: GTCACTGCCTTCGGTAAATAC
	F2: ATGCCAAGCCTTCGTTCTTTTA	
Primers used for transgenic Drosophila flies study		
CYP4S24	CCGGGATCCCAAAATGGATTTACTAACAATCAACAG	CTAGCTAGCATTGCCACGCGATTTTATG
CYP6A36	CCGAGATCTCAAAATGATCGCTTTGACAATA	CTAGTCTAGACAACTTCTCCACCTTCAA
CYP6A52	CCGAGATCTCAAAATGGTTTTTCTAACGCTT	CTAGTCTAGACAACTTCTCCACCTTCAA
CYP6D10	CCGGAATTCCAAAATGTTTTTATATTTGGCTATATTCG	CTAGGCTAGCACATCGTTTTATGAGTGTAATTTTC
Primers used for in vitro metabolism study		
CYP4S24	CACCATGGATTTACTAACAATCAA	TCAATTGCCACGCGATTTTA
CYP6A36	CACCATGATCGCTTTGACAATACT	TCAAATCCTCTCCACACGCA
CYP6D10	CACCATGTTTTTATATTTGG	TCAACATCGTTTTATGAGTGTAATTTTC
Reductase	CACCATGAGCGCGGAACAC	CTAGCTCCAAACGTCCGCG

Table S2. List of selected P450 genes in the ALHF *M. domestica*

Name[†]	SC_number[‡]	XM_number[‡]	XP_number[‡]
CYP18A1	NW_004765049	XM_005183375	XP_005183432
CYP304A2	NW_004765002	XM_005183057	XP_005183114
CYP305A1	NW_004764745	XM_005180589	XP_005180646
CYP306A1	NW_004765049	XM_005183378	XP_005183435
CYP28B1	NW_004764738	XM_005180394	XP_005180451
CYP28B2	NW_004764738	XM_005180398	XP_005180455
CYP28G6	NW_004765174	XM_005184255	XP_005184312
CYP310B2	NW_004765160	XM_005184125	XP_005184182
CYP317A3	NW_004765183	XM_005184339	XP_005184396
CYP438A4	NW_004765049	XM_005183376	XP_005183433
CYP6A1	NW_004765183	XM_005184331	XP_005184388
CYP6A24	NW_004768817	XM_005190469	XP_005190526
CYP6A25	NW_004768817	XM_005190472	XP_005190529
CYP6A36	NW_004765183	XM_005184332	XP_005184389
CYP6A37	NW_004765183	XM_005184336	XP_005184393
CYP6A4	NW_004765183	XM_005184338	XP_005184395
CYP6A40	NW_004765183	XM_005184343	XP_005184400
CYP6A5	NW_004765183	XM_005184346	XP_005184405
CYP6A52	NW_004765183	XM_005184334	XP_005184391
CYP6A54	NW_004760864	XM_005175565	XP_005175622
CYP6A57	NW_004768817	XM_005190468	XP_005190525
CYP6A58	NW_004765183	XM_005184341	XP_005184398
CYP6A59	NW_004765183	XM_005184344	XP_005184401
CYP6A63P	NW_004760864	XM_005175566	XP_005175623
CYP6A7	NW_004765183	XM_005184333	XP_005184390
CYP6C2	NW_004765183	XM_005184350	XP_005184407
CYP6D10	NW_004765160	XM_005184128	XP_005184185
CYP6D11	NW_004765015	XM_005183145	XP_005183202
CYP6D3	NW_004765160	XM_005184123	XP_005184180
CYP6D8	NW_004765436	XM_005185673	XP_005185730
CYP6EK2	NW_004765349	XM_005185208	XP_005185265
CYP6FS2	NW_004764906	XM_005182161	XP_005182218
CYP6FT3	NW_004768760	XM_005190452	XP_005190509
CYP6G4	NW_004766190	XM_005188667	XP_005188724
CYP6GU1	NW_004765183	XM_005184337	XP_005184394
CYP6GW1	NW_004768817	XM_005190471	XP_005190528
CYP6V3	NW_004764478	XM_005176346	XP_005176403
CYP9F10	NW_004764700	XM_005180062	XP_005180119
CYP9F11	NW_004764700	XM_005180054	XP_005180111
CYP9F12	NW_004764700	XM_005180063	XP_005180120
CYP9F7	NW_004764700	XM_005180052	XP_005180109
CYP9F8v1	NW_004764700	XM_005180050	XP_005180107
CYP9F9	NW_004764700	XM_005180053	XP_005180110
CYP3073A1	NW_004765739	XM_005187010	XP_005187067
CYP3073A2	NW_004765739	XM_005187012	XP_005187069
CYP3073B1	NW_004765739	XM_005187011	XP_005187068
CYP311A1	NW_004764740	XM_005180423	XP_005180480
CYP313D1	NW_004767316	XM_005189825	XP_005189882
CYP313D2	NW_004766063	XM_005188279	XP_005188336

CYP318B1	NW_004766506	XM_005189277	XP_005189334
CYP4AA1	NW_004764464	XM_005175977	XP_005176034
CYP4AC6v1	NW_004765632	XM_005186465	XP_005186522
CYP4AD1	NW_004765578	XM_005186278	XP_005186335
CYP4AE3	NW_004764514	XM_005177255	XP_005177311
CYP4C74	NW_004765515	XM_005185973	XP_005186030
CYP4D3	NW_004764514	XM_005177258	XP_005177315
CYP4D36	NW_004764744	XM_005180553	XP_005180610
CYP4D4	NW_004765144	XM_005183986	XP_005184043
CYP4D54	NW_004764514	XM_005177250	XP_005177307
CYP4D55	NW_004764514	XM_005177252	XP_005177309
CYP4D56	NW_004764514	XM_005177251	XP_005177308
CYP4D58v1	NW_004765144	XM_005183988	XP_005184045
CYP4D9	NW_004764514	XM_005177345	XP_005177402
CYP4E10	NW_004765578	XM_005186272	XP_005186329
CYP4E11	NW_004765578	XM_005186268	XP_005186325
CYP4E7	NW_004765578	XM_005186267	XP_005186324
CYP4G13	NW_004764475	XM_005176292	XP_005176349
CYP4G2	NW_004764475	XM_005176294	XP_005176351
CYP4G99	NW_004764542	XM_005177736	XP_005177793
CYP4P10	NW_004764771	XM_005180896	XP_005180953
CYP4P8	NW_004764771	XM_005180909	XP_005180966
CYP4S23	NW_004764524	XM_005177495	XP_005177552
CYP4S24	NW_004764524	XM_005177488	XP_005177545
CYP12A1	NW_004764697	XM_005180004	XP_005180061
CYP12A13	NW_004764697	XM_005180007	XP_005180064
CYP12A14	NW_004764697	XM_005179996	XP_005180053
CYP12A16	NW_004769267	XM_005190663	XP_005190720
CYP12A17	NW_004764512	XM_005177016	XP_005177073
CYP12A2	NW_004764697	XM_005179998	XP_005180055
CYP12A3	NW_004764697	XM_005179997	XP_005180054
CYP12G2	NW_004764745	XM_005180644	XP_005180701
CYP12G4	NW_004765031	XM_005183241	XP_005183298
CYP301A1	NW_004764517	XM_005177409	XP_005177466
CYP302A1	NW_004764628	XM_005179205	XP_005179262
CYP314A1	NW_004764603	XM_005178726	XP_005178783
CYP315A1	NW_004765301	XM_005184970	XP_005185027

† Nomenclature provided by the cytochrome P450 nomenclature committee, David R. Nelson.

‡ RefSeq accession number, National Center for Biotechnology Information, Bethesda, MD. SC_number: supercontig number; XM_number: mRNA sequence number; XP_number: amino acid sequence number.

Table S3. Relative expression profile of 86 P450 genes in three strains of *Musca domestica*

	Relative gene expression ± SEM*		
Gene	aabys	CS	ALHF
Expression significantly up-regulated in the ALHF house flies compared to both aabys and CS strains			
CYP4E10	1.00	1.56±0.08	274.42±22.40
CYP4E11	1.00	1.22±0.12	5.86±0.55
CYP4G13	1.00	0.99±0.11	2.13±0.13
CYP4G99	1.00	0.54±0.07	4.52±0.31
CYP4S24	1.00	1.04±0.11	2.94±0.08
CYP6A36	1.00	1.11±0.03	7.03±0.64
CYP6A40	1.00	1.08±0.11	2.95±0.22
CYP6A52	1.00	1.01±0.11	2.74±0.15
CYP6A58	1.00	0.84±0.18	4.32±1.02
CYP6D10	1.00	1.77±0.07	6.24±0.63
CYP6D3	1.00	0.26±0.05	2.40±0.13
CYP9F10	1.00	1.23±0.09	2.01±0.19
Expression significantly down-regulated in the ALHF house flies compared to both aabys and CS strains			
CYP12A3	1.00	0.74±0.01	0.35±0.15
CYP12G4	1.00	1.21±0.12	0.35±0.05
CYP28G6	1.00	1.18±0.17	0.21±0.04
CYP3073A1	1.00	1.03±0.11	0.12±0.03
CYP4AD1	1.00	0.94±0.08	0.07±0.01
CYP4D4	1.00	2.25±0.19	0.66±0.08
CYP6A24	1.00	1.08±0.11	0.69±0.07
CYP6A5	1.00	1.44±0.08	0.67±0.07
CYP6A57	1.00	1.89±0.17	0.66±0.03
CYP6A63P	1.00	0.22±0.05	0.08±0.03
CYP6D8	1.00	0.74±0.09	0.18±0.04
CYP6EK2	1.00	1.39±0.13	0.41±0.11
CYP6FT3	1.00	0.83±0.09	0.06±0.01
CYP6GW1	1.00	0.84±0.07	0.08±0.01
Others			
CYP12A1	1.00	1.28±0.12	1.02±0.19
CYP12A13	1.00	0.04±0.04	0.54±0.06
CYP12A14	1.00	0.36±0.06	0.74±0.08
CYP12A16	1.00	1.96±0.13	1.85±0.17
CYP12A17	1.00	3.13±0.11	2.81±0.24
CYP12A2	1.00	1.67±0.08	1.53±0.14
CYP12G2	1.00	2.99±0.25	2.16±0.19
CYP18A1	1.00	2.75±0.23	2.64±0.22
CYP28B1	1.00	0.95±0.11	1.04±0.12
CYP28B2	1.00	1.68±0.08	1.55±0.14
CYP301A1	1.00	0.78±0.08	0.69±0.07
CYP302A1	1.00	0.20±0.05	0.68±0.07

CYP304A2	1.00	0.15±0.03	0.74±0.16
CYP305A1	1.00	4.27±0.34	2.32±0.18
CYP306A1	1.00	0.95±0.10	1.09±0.11
CYP3073A2	1.00	0.34±0.06	0.37±0.05
CYP3073B1	1.00	0.74±0.13	0.88±0.09
CYP310B2	1.00	1.25±0.31	1.51±0.14
CYP311A1	1.00	0.96±0.11	0.86±0.09
CYP313D1	1.00	530.5±46.61	512.12±27.76
CYP313D2	1.00	0.93±0.12	0.79±0.08
CYP314A1	1.00	1.65±0.15	1.26±0.12
CYP315A1	1.00	1.88±0.13	2.07±0.18
CYP317A3	1.00	0.98±0.13	1.14±0.11
CYP318B1	1.00	0.91±0.13	0.99±0.11
CYP438A4	1.00	0.89±0.11	0.88±0.09
CYP4AA1	1.00	1.13±0.11	1.30±0.06
CYP4AC6v1	1.00	27.45±3.72	23.59±1.94
CYP4AE3	1.00	0.44±0.06	0.62±0.08
CYP4C74	1.00	5.16±0.41	1.60±0.15
CYP4D3	1.00	0.89±0.13	1.06±0.13
CYP4D36	1.00	1.11±0.08	1.03±0.12
CYP4D54	1.00	0.41±0.06	0.59±0.06
CYP4D55	1.00	0.53±0.07	1.04±0.16
CYP4D56	1.00	0.79±0.09	0.51±0.06
CYP4D9	1.00	1.02±0.11	1.08±0.13
CYP4D58V1	1.00	5.87±0.39	4.33±0.93
CYP4E7	1.00	1.11±0.11	1.05±0.12
CYP4G2	1.00	1.08±0.05	1.20±0.23
CYP4P10	1.00	2.98±0.26	2.93±0.11
CYP4P8	1.00	1.02±0.07	0.81±0.08
CYP4S23	1.00	11.69±1.31	6.61±0.55
CYP6A1	1.00	2.94±0.42	2.91±0.25
CYP6A25	1.00	3.67±0.32	1.05±0.10
CYP6A37	1.00	1.04±0.11	0.88±0.06
CYP6A4	1.00	2.02±0.18	1.18±0.05
CYP6A54	1.00	3.55±0.29	2.57±0.23
CYP6A59	1.00	1.17±0.12	0.86±0.09
CYP6A7	1.00	52.34±3.8	10.41±0.86
CYP6C2	1.00	1.51±0.14	0.82±0.08
CYP6D11	1.00	0.43±0.06	0.77±0.08
CYP6FS2	1.00	0.97±0.11	1.24±0.12
CYP6G4	1.00	3.22±0.26	2.41±0.31
CYP6GU1	1.00	1.79±0.16	1.91±0.21
CYP6V3	1.00	0.96±0.10	0.92±0.09
CYP9F11	1.00	1.24±0.05	0.99±0.09
CYP9F12	1.00	2.17±0.19	1.02±0.10
CYP9F7	1.00	1.23±0.09	1.31±0.12
CYP9F8v1	1.00	1.24±0.12	1.18±0.09

CYP9F9	1.00	1.06±0.11	0.81±0.08

* The relative levels of gene expression were shown as a ratio in comparison with that in aabys flies, the data are shown as the mean ± S.E.M.

Table S4. Ethoxycoumarin 7-O-deethylation activity of recombinant P450s/CPR proteins

	CYP4S24/CPR	CYP6A36/CPR	CYP6D10/CPR
7-Hydroxycoumarin formed (pmol/min/mg microsomal protein)	147.06± 7.11	262.55 ± 8.38	512.73 ± 5.44

Table S5. Docking results of selected P450 homology models

Enzyme	Active cavity volume (Å^3) §	Chemical	Putative metabolic site#	Estimate free energy (kcal/mol)	Distance from heme iron (Å) *
CYP6A36	482	cis-permethrin	gem	-8.31	2.94
			C5-PB	-7.92	3.23
			C4′-PB	-8.55	2.91
		trans-permethrin	gem	-10.93	2.90
			C5-PB	-9.21	3.13
			C4′-PB	-10.5	2.79
		PBalc	C4′-PB	-6.07	3.53
		Pbald	C4′-PB	-6.88	3.51
CYP6A52	537	cis-permethrin	gem	-8.83	3.36
			C5-PB	-7.24	3.62
			C4′-PB	-7.47	3.61
		trans-permethrin	gem	-10.13	2.91
			C5-PB	-8.52	3.42
			C4′-PB	-8.93	3.01
		PBalc	C4′-PB	-6.86	3.46
		Pbald	C4′-PB	-6.73	3.24
CYP6D10	574	cis-permethrin	gem	-8.72	3.52
			C5-PB	-8.06	3.60
			C4′-PB	-8.13	3.11
		trans-permethrin	gem	-9.70	2.92
			C5-PB	-9.05	3.35
			C4′-PB	-9.77	2.96
		PBalc	C4′-PB	-6.91	3.41
		Pbald	C4′-PB	-6.73	3.62
CYP4S24	279	cis-permethrin	gem	-7.95	4.93
		trans-permethrin	gem	-8.11	4.87
		PBalc	C-OH	-8.39	3.13
		Pbald	C-HO	-8.58	3.11

§ Active site cavity was calculated using the VOIDOO program with conventional probe radius of 1.4 Å. Å, angstrom. 1 Å=10^{-10} m

Predicted metabolic sites are indicated as following: Gem, germinal-dimethyl group; C5-PB, carbon 5 in alcohol moiety; C4′-PB, carbon 4′ in terminal aromatic ring; C-OH, carbon attached to hydroxyl group; C-HO, carbon of carbonyl group

* The distance between the heme iron and putative metabolic sites
